# Recent Advances in Defining the Immunoproteome of *Mycobacterium tuberculosis*

**DOI:** 10.3389/fimmu.2013.00335

**Published:** 2013-10-11

**Authors:** Shajo Kunnath-Velayudhan, Steven Anthony Porcelli

**Affiliations:** ^1^Department of Microbiology and Immunology, Albert Einstein College of Medicine of Yeshiva University, Bronx, NY, USA; ^2^Department of Medicine, Albert Einstein College of Medicine of Yeshiva University, Bronx, NY, USA

**Keywords:** peptide library, protein microarray, antigen discovery, type VII secretion system, ESX proteins, PE/PPE proteins

## Abstract

Immunity conferred by antigen-specific CD4+ T cells is critical for controlling infection with *Mycobacterium tuberculosis* (Mtb), the causative agent of tuberculosis. However, despite research that spans more than a century, many of the characteristics of protective immune responses to Mtb remain elusive. Defining the repertoire of antigenic targets is central to understanding the immune response against this pathogen. Although traditional methods of antigen discovery have identified many immunodominant antigens, they afford limited proteome coverage. Recent advances in proteomic techniques that are based on peptide library and protein microarray technology have enabled interrogation of the entire proteome of Mtb for antigens. Though these techniques have limitations and are still evolving, early studies using these techniques provide an unbiased view of the immune response to Mtb. Here we review proteome-wide approaches to antigen discovery and summarize what these have revealed so far on the composition of the Mtb immunoproteome.

## Introduction

Decades of research on T cell responses to *Mycobacterium tuberculosis* (Mtb) have identified many immunodominant antigens, some of which provide significant protection as immunogens in animal models (1). A major focus has been to identify antigens recognized by CD4+ T cells, since these cells are believed to play a dominant role in controlling Mtb infection (2). However, robust immune responses induced by immunodominant antigens do not necessarily translate into protective immunity (3), which reflects the complex and incompletely understood role of host immunity in the natural history of tuberculosis. On the one hand, antigen-specific CD4+ T cell responses are critical for protection against tuberculosis, and Mtb has evolved many strategies that subvert and evade the host adaptive immune response (4). On the other hand, Mtb exploits immune responses for its own benefit, and evidence suggests that immune-mediated tissue destruction facilitates the spread of Mtb among hosts. In agreement with this, recent studies have found that T cell epitopes of known immunodominant antigens of Mtb are hyperconserved, implying that immune responses against them may be in some cases more beneficial to the bacilli than to the host (5).

Given this complex relationship between host and pathogen, a full definition of the antigenic repertoire or “immunoproteome” of Mtb is an important step toward understanding how to effectively vaccinate against this infection. Until recently, most approaches to antigen discovery were based on traditional methods for separation and identification of antigens from complex mycobacterial protein mixtures (6). Alternative methods have employed screening of Mtb expression libraries in *Escherichia coli* with T cell clones derived from latently infected individuals (7). Although these methods have been successful in identifying immunodominant antigens, they provide restricted coverage of the Mtb proteome which has over 4,000 proteins. Advances in proteome-wide screening methods now enable a more extensive and unbiased survey of antigenic targets on complex pathogens like Mtb. Here, we review results from recently published proteome-wide antigen screens, and discuss how this powerful new approach may improve our understanding of the CD4+ T cell response to Mtb.

## Defining the Immunoproteome of Mtb

**Figure 1:**
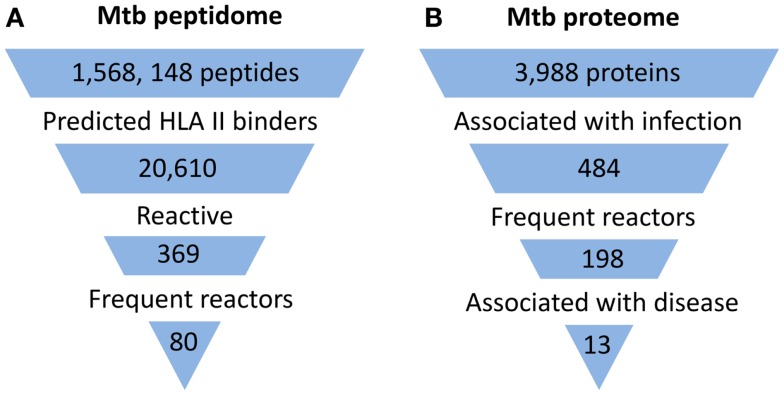
**Summary of proteome-wide screens for Mtb antigens**. **(A)** Summary of screen for targets of CD4+ T cells (8). Mtb peptide sequences that represented 5 complete and 16 incomplete Mtb genomes were analyzed by HLA Class II consensus prediction method for binding to 22 of the most commonly expressed alleles of HLA-DR, -DP, and -DQ sequences. Peptides predicted to bind with high affinity were synthesized and tested by ELISPOT for stimulation of IFNγ production by circulating T cells of 28 latently infected, non-BCG vaccinated donors from a non-endemic area. Among the 369 reactive peptides, 80 peptides accounted for 75% of the total response. **(B)** Summary of one of two published screens for targets of humoral responses (11). Approximately 95% of the open reading frames of Mtb (H37Rv strain) corresponding to 3,988 proteins were cloned and expressed *in vitro* in an *Escherichia coli*-based cell-free transcription/translation system. The crude reactions containing expressed proteins were printed directly onto nitrocellulose-coated slides without purification. These slides were then probed with sera from uninfected healthy individuals from a non-endemic country (*n* = 64) and suspected cases of TB (TB and non-TB pulmonary patients) from endemic countries (*n* = 561). The proteins that reacted to sera from endemic countries but not to sera of uninfected individuals were defined as antigens associated with infection. Among 484 such antigens, 198 reacted to more than one serum from endemic countries and were designated frequent reactors. Proteins associated with disease were identified by comparing responses in TB patients and non-TB patients.

With the development of technologies that allow high-throughput peptide and protein synthesis, it is now possible to interrogate the entire Mtb proteome for antigens. So far, three proteome-wide and relatively unbiased approaches to identify candidate antigens for CD4+ T cell responses from the Mtb proteome have been described (Figure 1). One approach was based on the use of a peptide library designed to screen potential targets of CD4+ T cell responses in latently infected individuals (8). In contrast to previous studies which typically involved *in vitro* expansion of Mtb-specific T cells, circulating T cells from Mtb infected donors were directly tested against the synthetic peptide library using IFNγ ELISPOT assay. This screen involved predicting Mtb peptides that bind with high affinity to commonly expressed MHC class II alleles using a consensus approach based on results from three prediction methods (9). Two other studies used analysis of serum antibody responses as a surrogate for CD4+ T cell responses, relying on the assumption that a strong linkage exists between the targets of antibodies and of the CD4+ helper T cells involved in their generation (10). In one case, protein microarrays printed with products of all expressed open reading frames of Mtb were used to screen sera from TB patients and controls for antibody reactivity (11). In the other study, a similar screen for serum antibody responses against the Mtb proteome in TB patients was performed using traditional methods of recombinant protein expression and ELISA (12).

Despite major differences in their goals and methodology, all studies concluded that human immune responses target a subset of the Mtb proteome during infection (Figure 1). In the direct screen for CD4+ T cell responses to synthetic peptides, ∼2% of the proteome accounted for 80% of the total responses (8). With the serum antibody-based screens, a larger fraction of the proteome was found to be immunogenic, constituting ∼6–10% of the proteome (11, 12). In part, this difference may reflect that the latter two studies screened for immune responses in active TB patients, who would generally be expected to have higher bacterial burdens than the latently infected individuals studied by Lindestam Arlehamn et al. A striking feature of all studies was that the subset of the proteome that induced an immune response (i.e., the immunoproteome) was enriched for secreted and cell wall-associated proteins. This is consistent with earlier studies which have suggested that the immunodominant antigens of mycobacteria are proteins that are secreted by the bacteria during infection, and also with the recent finding that mycobacteria secrete membrane vesicles rich in antigens (13). Although the components of the immunoproteome varied and showed only partial overlap among these studies, a consistent finding was the remarkable prominence of immune responses directed at members of the PE/PPE and ESX protein families, which are well known as critical virulence determinants of mycobacteria.

## PE/PPE Proteins as Targets of CD4+ T Cells

**Figure 2:**
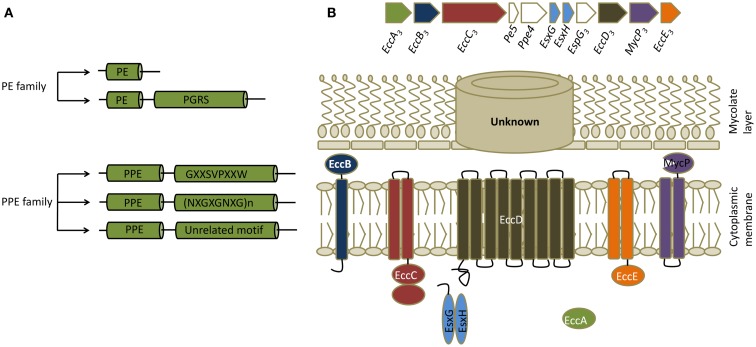
**PE/PPE and ESX proteins**. **(A)** Classification of PE/PPE proteins. PE/PPE proteins are broadly divided into PE and PPE proteins based on their characteristic N-terminal proline-glutamic acid (PE) and proline-proline-glutamic acid (PPE) sequences. These motifs occur within a span of ∼110 aminoacids in PE proteins and ∼180 aminoacids in PPE proteins. The PE family is further subdivided into two groups based on the presence or absence of a C-terminal domain with multiple tandem repeats of Gly-Gly-Ala or Gly-Gly -Asn sequences (PGRS, polymorphic GC-rich repetitive sequences). The PPE family is subdivided into three groups based on characteristic motifs in their C-termini, as indicated. **(B)** General features of ESX secretion systems. Organization of genes in the ESX-3 cluster, which induced maximal responses in the CD4+ T cell screen, are shown along with a schematic of a typical ESX or type VII secretion system. All ESX clusters contain a pair of *Esx* genes, the products of which form 1:1 complexes that are secreted. ESX clusters can exist as either complete (designated ESX-1 through ESX-5 systems) or partial clusters. In addition to the two *Esx* genes, complete ESX clusters encode four core components (ESX core component, *Ecc*) which are *EccA* (an ATPase), *EccB* (a membrane protein), *EccC* (an ATPase), *EccD* (a transmembrane protein), *EccE* (a transmembrane protein), and *EccF* (MycP, a subtilisin-like serine protease). Based on the current model of type VII secretion system, Esx heterodimers are recognized by EccC which then form an active ATPase providing energy for transport. EccC then propels these substrates through EccD, the transmembrane protein forming the export channel. MycP might be involved in processing certain substrates. The channel that transports the substrates thought the mycolate layer is yet to be identified. ESX clusters contain additional genes that code for other proteins called ESX secretion-associated proteins (*Esp*). In most of the complete and incomplete ESX systems, a pair of genes that code for PE/PPE proteins also exists in close proximity to the *Esx* genes.

PE/PPE proteins are unique proteins found exclusively in pathogenic mycobacteria. There are genes for 99 PE proteins and 68 PPE proteins in the genome of Mtb, constituting ∼10% of its coding capacity (14). They are characterized by the presence of proline-glutamic acid (PE) and proline-proline-glutamic acid (PPE) motifs near their N-termini (15). These proteins are further divided into subfamilies based on the motifs in their C-termini (Figure 2). Many PE/PPE proteins are localized to the bacterial cell surface and many are secreted, making them readily accessible to the immune system (16). Indeed, PE/PPE proteins are known to induce strong humoral and cellular immune responses based on studies that used traditional immunological assays (17). In the genome, PE/PPE genes are often found within operons or gene clusters that also contain a variety of other types of genes. Most notably, genes for some PE/PPE proteins map within gene clusters encoding type VII secretion systems, which are known as ESX systems in mycobacteria (see below). Secretion of some of the PE/PPE proteins is known to be dependent on ESX systems, and comparative genomics studies suggest that ESX clusters have coevolved with PE/PPE proteins. The role of this distinct family of proteins in mycobacterial virulence is yet to be determined, although studies suggest involvement in many aspects of pathogenesis including bacterial attachment to host cells, immunomodulation, and ability to persist in granulomas (15, 17).

All three published proteome-wide antigen screens showed that PE/PPE proteins are major targets of human immune responses. Among the antigens identified by peptide library screening for CD4+ T cell responses, ∼45% were PE/PPE proteins though they constitute <5% of the open reading frames of Mtb. In addition, PE/PPE proteins represented approximately half of the novel antigens identified, suggesting that immune responses to these proteins remain mostly unexplored. Proteome-wide antibody screening also revealed an enrichment of PE/PPE proteins among the antigens though the enrichment was less than that observed for CD4 responses (an average of ∼5–6% of the total targets recognized). It is possible that the relatively lower enrichment found for these proteins for antibody responses compared to cellular responses could in part reflect difficulties in expressing these proteins by recombinant techniques that were used to generate antigens for the antibody-based screening. The identification of PE/PPE proteins as prominent antigenic targets of CD4+ T cells in these studies of the immunoproteome is consistent with other more targeted analyses showing that these proteins are highly immunogenic in mycobacteria-infected humans and cattle (18).

## Components of ESX Secretion System as Targets of CD4+ T Cells

ESX secretion systems are specialized protein export systems originally identified in mycobacteria and constitute a distinct mechanism for protein secretion known as the type VII secretion system. The system is named after two proteins, EsxA (also known as Early Secretory Antigenic Target-6, or ESAT-6) and EsxB (Culture Filtrate Protein-10, or CFP-10). There are five complete (ESX-1–5) and five to six incomplete ESX secretion systems in Mtb, and all carry a pair of genes that encode homologs of EsxA and EsxB (Figure 2). Most of the complete and incomplete ESX systems also contain or are closely linked to a pair of genes that code for PE/PPE proteins. These secretion systems export small proteins that contain a WXG amino acid motif (tryptophan and glycine separated by one amino acid). Secretion of EsxA and EsxB or their homologs, as well as many PE/PPE proteins, is dependent on ESX systems. Despite similarities, ESX systems do not complement each other and each one is likely to play distinct roles in Mtb virulence and physiology. For example, ESX-1 is required for survival of mycobacteria in mice, for granuloma formation, and for escape of Mtb from phagosome into cytosol (19, 20). Similarly, ESX-3 is involved in iron and zinc uptake by the bacteria, while ESX-5 has a role in modulating immune responses (20). It should be noted that these systems and their homologs are found in pathogenic and non-pathogenic mycobacteria and in other gram positive bacteria (21).

ESX proteins are among the well known immunodominant antigens of Mtb. Commercial tests for Mtb infection (QuantiFERON^®^ and T-SPOT.TB) use EsxA and EsxB as antigens since circulating T cells from most infected individuals respond strongly to these proteins. However, the proteome-wide screens reveal for the first time the extent of immunodominance of ESX proteins. For example, Lindestam Arlehamn et al. showed that 42% of the cellular responses they detected were directed against a set of proteins that represented 0.55% of the Mtb genome. These proteins were encoded by genes located in three distinct regions of the Mtb genome, which the authors called antigenic islands. All three islands include Esx protein pairs (i.e., EsxA/EsxB or homologs) and two contain the complete type VII secretion systems ESX-1 and ESX-3. Immunoproteome analysis using antibody screening also revealed that components of ESX systems induce strong immune responses in humans. Pathway analysis of the data from probing of microarrays with sera from active TB patients showed a significant enrichment in reactivity to proteins controlled by Zur, a regulator of Zinc uptake. Zur regulates a set of 56 genes which includes genes that code for three ESX systems. Interestingly, two of these three systems (ESX-3 and a partial ESX cluster containing genes for EsxQ, EsxR, and EsxS) were included in the three antigenic islands identified in the proteome-wide screen for CD4+ T cell responses. In addition, two proteins of the ESX-1 system were among the top five antigens associated with active TB. Collectively, these data underscore the immunodominance of ESX systems.

Surprisingly, it appears that both secreted and non-secreted components of the ESX systems induce strong immune responses. For example, previous studies have shown that eight different T cell antigens are encoded by genes in and around the ESX-1 region. Since the antigens tend to cluster in the genome, ESX systems had been called immunogenicity islands (22). It is not clear how the non-secreted components of the ESX secretion systems induce strong immune responses compared to other unrelated, surface-associated proteins. Since homologs of ESX systems exist in many bacteria, it is possible that cross-reactive epitopes may contribute to enhanced recognition of non-secreted components. Co-regulation might also explain some of the immunodominance. For example, bacteria may overexpress secreted and non-secreted components of the ESX-3 system in conditions of low iron or zinc. In addition, the components of these systems might exist as multi-protein aggregates, as shown for the ESX-5 system (23). This could favor the spreading of immune responses from epitopes of the secreted components to epitopes of the non-secreted components. Another possibility is that the secretion system may form structures protruding from the bacterial cell surface, thus increasing accessibility to antigen processing machinery (19). Although the immunoproteome analysis suggested that ESX proteins, and less strikingly PE/PPE proteins, in the antigenic islands were more immunogenic than their counterparts encoded in other areas of the genome (8), other more focused studies in *M. bovis* infected cattle have not confirmed this finding (18, 22, 24).

## Limitations of Current Methods for Proteome-Wide Screening

Although uniquely powerful, high-throughput approaches of antigen discovery do not necessarily capture responses to all immunodominant antigens. For example, in the screen for cellular responses, only 20–25% of the latently infected donors responded to EsxA or EsxB (8). These antigens are known to induce responses in most latently infected individuals, which is the basis for their use in commercial *ex vivo* tests for latent infection. Similarly, in another analysis of 18 latently infected individuals based on responses to peptide libraries (25), no responses were detected to the TB7.7 antigen. This antigen is also used in a commercial test for latent infection and stimulates responses in approximately half of infected individuals (26, 27). Similarly, proteome-wide screens based on expression of recombinant proteins may yield false negative results because of failure to express certain Mtb proteins. Indeed, the microarray-based screen for humoral responses failed to detect some known immunodominant antigens, importantly EsxA (11). In addition, the screen by Li et al. failed to detect responses to both EsxA and EsxB. Another limitation is that pre-selection of peptides that are predicted to bind to MHC class II molecules by Lindestam Arlehamn et al. may have excluded some immunodominant peptides, since the performance of the predictive algorithms used is known to suffer from a significant rate of both false positive and false negative errors (9).

## Conservation Versus Variation of Epitope Sequences

The question of whether certain immune responses induced by Mtb antigens could actually be more beneficial to the bacteria than to the host remains open. According to classical models that portray the host-pathogen interaction as an “evolutionary arms race,” immunodominant epitopes are likely to be less conserved due to high selection pressure from the immune response. Recent studies suggest that this model may not consistently apply to mycobacteria. By analyzing genome sequences of a diverse collection of Mtb strains, Comas et al. showed that the great majority of known T cell epitopes display little sequence variation, with lower ratios of non-synonymous to synonymous changes than seen in other coding regions of essential and non-essential genes (5). Though the study by Comas et al. excluded members of PE/PPE protein family for technical reasons, a recent study of Mtb isolates showed that PE/PPE proteins are also not under diversifying selection pressure (28). One caveat of these studies is that they used epitope sequences that were originally identified from studies that used the genomic sequence of a single laboratory strain of Mtb (H37Rv), and this approach would be expected not to include epitopes that are highly variable among random clinical isolates. Thus, the analysis may have been inadvertently restricted to invariant epitopes, leading to severe biasing of the results. In addition, use of a small number of antigens might have caused further bias, as approximately two thirds of the epitopes in the database used to design these studies (the Immune Epitope Database) are derived from only thirty antigens (29). In contrast to the studies showing conservation of epitope sequences, another study has shown evidence for sequence variation in known T cell epitopes suggesting that they may diversify in order to evade immune recognition (30). Thus, a clear and consistent picture of the level of epitope conservation versus variation has yet to be established.

For antigens that derive from multigene families such as the PE/PPE and ESX proteins, another potentially important feature that may contribute to immunodominance is the presence of cross-reactive epitopes that are shared by multiple homologous family members. Indeed, PE/PPE proteins are known to carry highly conserved sequences. The relevance of this point was suggested by a study showing that both magnitude and frequency of T cell responses to PE/PPE proteins were greater to peptide pools representing the more conserved N-terminal regions than to pools representing other regions of the proteins (18). In addition, a majority of the reactive peptides showed more than 70% sequence similarity to one or more additional PE/PPE regions, further suggesting that cross reactivity contributes significantly to responses to PE/PPE proteins. Another study which examined responses to ESX-5 encoded PE/PPE proteins in mice also concluded that cross-reactive epitopes contribute significantly to the immunodominance of PE/PPE proteins (31). However, in contrast to the response to PE/PPE proteins, analysis of bovine responses to ESX proteins did not reveal an association between immunodominance of specific epitopes and their level of cross reactivity with other homologous Esx proteins (24).

## Conclusion

New methods enabling proteome-wide antigen screens provide for the first time a general view of the immune responses induced by Mtb. The data obtained so far using these methods indicate that during Mtb infection, immune responses target a small subset of the proteome enriched for membrane-associated and secreted proteins. Among these antigens, members of the families of PE/PPE and ESX proteins are major targets of immune responses. These results should encourage future efforts to characterize the role of these protein families in pathogenesis, and stimulate interest in developing them as components of novel vaccines. While these two protein families comprise important potential components of novel vaccines, it remains to be determined whether the most prominent antigens in natural infection are also among the most effective antigens in the context of vaccination. Since most of the data obtained so far have come from analyses of immune responses in chronically infected individuals, it will be important to extend these studies to subjects with acute infection who may target a different antigenic repertoire.

## Conflict of Interest Statement

The authors declare that the research was conducted in the absence of any commercial or financial relationships that could be construed as a potential conflict of interest.
